# Cases of Hydrophobia, with the Appearances on Dissection, and Observations on the Means of Prevention

**Published:** 1807-03

**Authors:** M. Sabatier

**Affiliations:** Surgeon to the Hotel des Invalides, at Paris


*Cases of Hydrophobia, with the Appearances on Dissection, and Observations on the Means of Prevention;* by M. Sabatier, Surgeon to the Hotel des Invalides, at Paris.

In 1784, I communicated to the Academy of Sciences the case of a patient bitten by a mad dog in several parts of the body. In this case the wounds were cauterized by means of muriate of antimony, and the patient remained exempt from the disease. In the same communication I cursorily mentioned two patients, who in 1775 had, under my care, been treated in the same manner, with similar success; as well as two others, who in 1780 had each a phalanx of one of their fingers amputated, in consequence of being injured by a rabid animal.

Since that period, only a single instance has occurred in my practice, wherein cauterization was employed. This patient was treated, as in the first case, by muriate of antimony, and the result proved equally favourable. I am sensible that no positive conclusion can be deduced from such a limited number of cases; yet if they be taken in conjunction with that of 1784, and those of the late M. Le Roux, an eminent surgeon at Dijon, which are inserted in the Memoirs of the Society at that place, it must, I think, be admitted that they afford a strong presumption in favour of this practice, as a preventive of rabid hydrophobia.

Besides the above cases, in which the patients happily remained free from the disease, others have fallen under my observation, wherein similar preservative means were not not attended with equal success, or in which particular circumstances prevented their application. Conceiving that a more full account of the cases referred to above, as well as a detail of those which terminated fatally, with the appearances on dissection, might tend to throw farther light on the nature of this formidable malady, I have been hence induced to lay them before the public.

On the evening of the 18th of August, 1766, a soldier belonging to a corps of invalids, when on guard before the porch of the Hotel de la Guerre at Versailles, being wrapped up in his watch cloak, with his arms crossed over his breast, perceived a dog seize the bottom of his cloak behind, which it continued to hold for a considerable time. Irritated at such an attack, he endeavoured to shake off the animal, on which it sprang upon his breast, and bit him in the face. His musquet, which was standing against the wall, was thrown down, but he drew his sword, in order to defend himself, on which the animal took to flight. The wounds were inflicted on the exterior angle of the right eye, and near the corner of the lips on the same side. They bled profusely, but healed in a short time, without producing afterwards any inconvenience.

The apprehensions he expressed in consequence of this accident, induced him to have recourse to a celebrated prophylactic medicine, sold at that time in the village of Viroflay. This medicine was directed to be taken during nine days, but his officers in the mean time sent him to the Hotel des Invalides. The beneficial effects attributed to mercurial frictions, as a preventive against Rabies Canina, induced me to have recourse to them on the present occasion. After bleeding, and the exhibition of a cathartic, he was ordered to rub in a portion of mercurial ointment daily, and at the same time to employ the warm bath. He was twice purged towards the termination of this course, which was persevered in till the 15th of September. During its continuance, two ounces of strong mercurial ointment were employed.

Hitherto the patient remained free from every complaint, except experiencing slight attacks of vertigo. He had a good appetite, and his countenance had undergone no change. On the 16th, he complained of a weight in his head, and his features were considerably altered. He passed a restless and unquiet night. I prescribed for him a bolus composed of vitriolated quicksilver and zedoary root, in the quantity of four grains each, in a little theriaca, with a few glasses of a sudorific decoction. By these

 remedies, remedies, which were repeated three successive days, the bowels were kept moderately open; the head-ach and vertigo disappeared, but his appetite was considerably diminished. His countenance had now experienced a very manifest change, and his nights were rendered unquiet by frightful dreams.

25th. He was attacked with violent spasms in the face and throat on attempting to drink. I ordered him a bolus composed of assafœtida, musk, and red sulphurated oxide of mercury, to be taken every three hours, and at the same time enjoined his throat to be carefully rubbed with a volatile liniment.

26th. His horror at liquids became more marked, and he complained of a sense of suffocation on attempting to drink; he supported, however, without any painful feelings, both the sight and noise of water. His eyes were haggard, and the pulse slow and feeble. The bolus was continued, with the addition of one grain, and afterwards of two grains of opium, and his drink was an infusion of the flowers of the lime tree. Although his horror at liquids still continued, it appeared somewhat diminished, and he even forced himself to swallow a little of the infusion.

27th. He evinced a frequent inclination to vomit, which induced me to order him four grains of tartarized antimony, dissolved in as many small glasses of water. This medicine produced a slight degree of vomiting, and afterwards proved cathartic. In the afternoon the difficulty of swallowing augmented. The prescription of yesterday was continued. He now appeared greatly incommoded by the sight of water; his pulse became hard, and increased in frequency; his voice was hoarse, and his respiration oppressed.

28th. The symptoms continued the same as yesterday. The patient still retained the power of swallowing, he even indicated a desire for a little soup, of which he got over a few spoonfuls. Towards evening, however, he experienced violent spasms in the head and neck, and was attacked with universal rigors. These symptoms recurred on the least noise; and the imagination of the patient was now incessantly haunted with the dread of death.

29th. He became delirious, his voice was more hoarse than on the preceding day, and his respiration more oppressed. He could still, however, swallow a little liquid, but with extreme difficulty. Towards the morning, and throughout the whole of the day, he ejected much saliva, every moment squirting it to the distance of more than twelve twelve feet, so that the attendants were obliged to keep the curtains of his bed shut, and not to approach it without the greatest precaution.

About four o'clock in the afternoon, the patient ceased to take any thing whatever; the delirious paroxysms were less violent, and he was not in the least degree furious. This patient never foamed at the mouth, or evinced the smallest disposition to bite. He died at six o'clock in the evening, while endeavouring to eject saliva.

Never having previously witnessed a case of rabid hydrophobia, I felt some reluctance to open the body, in which feeling my pupils in a certain degree participated. Having therefore requested the assistance of some of the pupils of the Hospice de l'Humanitie, who were in the habit of dissecting bodies, I acquired confidence from their example, and proceeded to assist them. The brain, thoracic, and abdominal viscera appeared in their natural state. None of the organs contained in this last cavity exhibited any appearance of those spasmodic contractions, so frequently observable in other cases. Neither did the fauces, or the œsophagus display the least marks of inflammation, or strangulation. These parts were of a greenish colour, which I attributed to a portion of the assafœtida contained in the bolusses having been dissolved by the saliva, in the act of swallowing them. It may, perhaps, be deemed somewhat singular, that although the patient had taken a considerable quantity of musk, his body and clothes preserved only the odour of the assafœtida. As a matter of precaution, I caused the walls of the ward to be white washed, and ordered the clothes worn by the deceased to be burnt.

From this period, about eight years elapsed before I witnessed another case of hydrophobia. This case occurred on the 19th of March, 1774. Nearly three months before, that is, on the 24th of December preceding, a subaltern officer on guard, during the night, behind the Dome of the Hotel des Invalides, was bitten in the fore finger of the right hand by a strange dog. He did not mention the accident, deeming it of no importance, and continued to live in his usual manner.

On the 19th of March, the day already mentioned, he came to the Infirmary, about five o'clock in the evening, after having passed a restless perturbed night; his complaints had not yet, however, assumed any marked character. I ordered a proper regimen and caused blood to be taken from the arm. The patient observed to one of my pupils, who was directed to perform this operation, that he found himself much oppressed; and, in fact, his breathing was quick, laborious, and accompanied with convulsive twitches. He experienced great agitation when drink was presented to him, and on bringing the vessel near his lips, dashed the liquid contained in it to a considerable distance. Shortly afterwards, on attempting to swallow a small quantity of water, he was affected with more violent spasms than any of those he had formerly experienced. He had not yet made any effort to throw out any saliva. This symptom, however, occurred on the following morning, about ten o'clock, and continued until his death, which happened on the evening of the same day. During this time, he foamed a little at the mouth, his body was bedewed with a cold and clammy sweat, and his countenance was dreadfully distorted. Every attempt to drink produced in his frame an universal shuddering. The groans, which he frequently uttered, somewhat resembled the howlings of a dog suffering under extreme pain, and his death was preceded by a total loss of consciousness.

No unusual appearances presented themselves on the examination of the body of this patient, either in the fauces, œsophagus, or stomach; a sort of roughness was observed in the superior portion of the œsophagus, which was white, and destitute of every sign of inflammation; the abdomen was very much inflated.

The next case of this kind, which fell under my observation, was that of 1775, already mentioned. On the night between the second and third of September, a strange dog had found his way into the Hotel des Invalides, and was observed, about midnight, by a subaltern officer on duty, ascending the stairs leading to the Governor's House. He shouted with the intention of frightening away the animal, on which, it sprung upon him, though at the distance of twelve feet. Its teeth penetrating through his shoulder-belt and clothes, he was wounded a little below the left breast towards the arm pit, on which he struck the dog over the stairs with the butt end of his musquet. A soldier sleeping upon the grass, in the large court, was attacked, about two hours afterwaids, by the same animal, which bit him in the crown of the head, and towards the edge of the left eye, carrying away a portion of the upper eye-lid, and a part of the upper lip. The cries of the soldier were heard by the subaltern officer, who scarcely recollected the accident that had befallen himself; besides, he durst not not leave his post. About day break, this dog entered another court, where, after biting several hogs, chickens, and ducks, he was killed by a servant, who luckily himself received no injury.

In the morning, these accidents made much noise in the Hotel; and about eleven o'clock, the officer came to me, and related what had happened. I advised him to repair to the Infirmary, where I immediately followed, and having placed him in a proper position, I cauterized the wound by means of burning tinder, which I retained on the part with a small brass wire, until an eschar was produced about the size of a shilling.

The next day, some individuals proposed setting on foot a subscription to enable the patients to proceed to the sea for the purpose of bathing. But the Directors of the Hotel on being informed of this circumstance, were of opinion that the expence ought to be defrayed from the funds of the institution, and addressed a memorial to the minister of War, in order to obtain his concurrence, which was readily granted.

I offered my services on the present occasion; and preparations having been hastily made, we set out on our journey, about three o'clock in the afternoon, on the same day, the patients in one carriage, and myself in another, accompanied by a stout intelligent domestic, who was capable of affording me proper assistance, in case of necessity. As the patients were much fatigued, we stopped a few hours at Rouen, and arrived at Dieppe on the third evening after the accident.

The landlady of the inn where we alighted, informed us that many people suffering under similar accidents resorted to this place; that immersion was here conducted in a peculiar manner, and only by people properly authorized to perform it.

She accordingly sent to me a man having the appearance of a sailor, who confirmed the account she had given, adding, that before the expiration of an hour, he would finish the operation; and I might then depart, as it was only necessary to perform it once. On his return, in a short time, with three of his companions, I accompanied them to the sea side, being curious to witness this superstitious practice. They immediately proceeded to undress the patients, not even suffering them to retain a tie on their hair, or a ring on their fingers; two of these men then grasped each patient by the elbows, and below the armpits, and made them proceed backward upon the beach

 until until the water reached up to their middle. Their conductors, whose own faces were turned towards the sea, threw them backwards on the approach of a wave; repeating this operation five times in the same manner. At first, the surprise which they experienced, made them cry out; they said nothing, however, on the subsequent immersions, but seemed considerably stunned, on being brought up for the fifth time. The bathers then rubbed and assisted them to dress, after which we returned to the inn.

On enquiry, one of these men informed me, that they were six in number, and authorized to carry on this profession by the municipality of Dieppe; so great was their credulity, that they entertained no doubts respecting the efficacy of the practice, and unhesitatingly affirmed, that no person, who had submitted to it, ever became affected with the disease.

I was fully convinced that no security could be afforded by this absurd practice; if the patients had either been suddenly thrown from a great height into the sea, or been kept under water till deprived of consciousness, or even if the bath had been regularly persevered in for a considerable length of time, some effect might have been perhaps supposed to ensue; but the operation in question being extremely momentary, and attended with no circumstances calculated to make a forcible impression on the imagination, how could it be expected to prove useful?

On my return to Paris, it was my intention to have administered to my patients strong doses of the Chinese remedy, which is composed of opium, red sulphurated oxide of mercury, and musk, employing at the same time a salt water bath: but the physician of the Hotel des Invalides, having taken the patients under his own immediate care, put them on a course of volatile alkali, and ordered the employment of a fresh water bath. I remained a quiet spectator of the method which he pursued, taking care to keep open for some time longer the wound in the subaltern officer's side, by means of a pledget soaked in a strong solution of concrete pot ash. From this period, (forty days after the accident) nothing more was attempted, and the physician having discontinued the ammoniacal medicine, the patients left the Infirmary, and returned to their usual manner of life.

On Tuesday the 25th of October following, the soldier, whose wounds I did not think it safe to cauterize at the time the accident happened, on account of their number and and position, returned to the Infirmary with the view of taking a medicine which was prescribed for him, in consequence of an indisposition, of which the true cause was not suspected. After its operation he withdrew to his apartment, and throwing himself upon his bed, he remained without food until the next morning, when he was brought back to the Infirmary.

( To be continued. )

